# Methods for designing interventions to change healthcare professionals’ behaviour: a systematic review

**DOI:** 10.1186/s13012-017-0560-5

**Published:** 2017-03-04

**Authors:** Heather L. Colquhoun, Janet E. Squires, Niina Kolehmainen, Cynthia Fraser, Jeremy M. Grimshaw

**Affiliations:** 1 Department of Occupational Science and Occupational Therapy, University of Toronto, 160-500 University Ave, Toronto, Ontario M5G 1V7 Canada; 2 Ottawa Hospital Research Institute, Clinical Epidemiology Program, The Ottawa Hospital, General Campus, 501 Smyth Road, Centre for Practice Changing Research, Ottawa, Ontario K1H 8L6 Canada; 3 School of Nursing, University of Ottawa, 451 Smyth Road, Ottawa, Ontario K1H 8M5 Canada; 4 Institute of Health and Society, Newcastle University, The Baddiley-Clark Building, Richardson Road, Newcastle upon Tyne, NE2 4AX UK; 5 Health Services Research Unit, University of Aberdeen, Health Sciences Building Foresterhill, Aberdeen, AB25 2ZD Scotland; 6 Department of Medicine, Epidemiology and Community Medicine, University of Ottawa, 451 Smyth Road, Ottawa, Ontario K1H 8M5 Canada

**Keywords:** Knowledge translation, Systematic review, Intervention design, Methodology

## Abstract

**Background:**

Systematic reviews consistently indicate that interventions to change healthcare professional (HCP) behaviour are haphazardly designed and poorly specified. Clarity about methods for designing and specifying interventions is needed. The objective of this review was to identify published methods for designing interventions to change HCP behaviour.

**Methods:**

A search of MEDLINE, Embase, and PsycINFO was conducted from 1996 to April 2015. Using inclusion/exclusion criteria, a broad screen of abstracts by one rater was followed by a strict screen of full text for all potentially relevant papers by three raters. An inductive approach was first applied to the included studies to identify commonalities and differences between the descriptions of methods across the papers. Based on this process and knowledge of related literatures, we developed a data extraction framework that included, e.g. level of change (e.g. individual versus organization); context of development; a brief description of the method; tasks included in the method (e.g. barrier identification, component selection, use of theory).

**Results:**

3966 titles and abstracts and 64 full-text papers were screened to yield 15 papers included in the review, each outlining one design method. All of the papers reported methods developed within a specific context. Thirteen papers included barrier identification and 13 included linking barriers to intervention components; although not the same 13 papers. Thirteen papers targeted individual HCPs with only one paper targeting change across individual, organization, and system levels. The use of theory and user engagement were included in 13/15 and 13/15 papers, respectively.

**Conclusions:**

There is an agreement across methods of four tasks that need to be completed when designing individual-level interventions: identifying barriers, selecting intervention components, using theory, and engaging end-users. Methods also consist of further additional tasks. Examples of methods for designing the organisation and system-level interventions were limited. Further analysis of design tasks could facilitate the development of detailed guidelines for designing interventions.

**Electronic supplementary material:**

The online version of this article (doi:10.1186/s13012-017-0560-5) contains supplementary material, which is available to authorized users.

## Background

Our project sought to advance the methods for translating research knowledge into practice. Knowledge translation (KT) is ‘a dynamic and iterative process that includes the synthesis, dissemination, exchange and ethically sound application of knowledge to improve health, provide more effective health services and products and strengthen the healthcare system’ [1]. One of the critical aspects of KT is that it requires healthcare professionals (HCPs) to change practice [2].

HCPs’ practice can be influenced by a wide range of factors; for example, a recent review identified 57 clusters of factors [3]. Specific interventions range from interventions targeted at HCPs (e.g. educational materials, audit and feedback) to interventions targeted towards consumers and policy-makers. The evidence base for many of these interventions remains incomplete [4], and there is an on-going need to design more effective interventions.

Systematic reviews of KT interventions to change HCPs’ practice consistently indicate that interventions are haphazardly designed and poorly specified, limiting our ability for replication, understanding, and generalizability [5, 6]. Limitations in intervention design impede evaluations of interventions [7, 8]. One issue contributing to the shortcomings in intervention design is a lack of agreed, practical ‘how to’ guidance for designing KT interventions.

Recommendations have been made to ensure that intervention design includes an assessment and prioritization of barriers, identification of potential adopters and practice environments, and consideration of both the potential effectiveness and feasibility of the chosen strategies [2], but these recommendations do not necessarily provide an approach to the design or development of the intervention [9]. Various potential tools (e.g. Behaviour Change Technique (BCT) taxonomy [10]) and sources describing a range of methods for mapping barriers and facilitators to KT interventions exist [11], but sources describing a range of complete methods for intervention design are few.

The aim of the present study was to contribute to the design of such a resource by synthesising literature about methods for designing KT interventions. Our specific objective was to systematically identify published methods for designing interventions to change HCPs’ behaviour.

## Methods

A systematic review was undertaken. We did not publish a protocol. The initial literature search included MEDLINE, Embase, and PsycINFO from1996 to April 2013. An identical search was conducted on April 20, 2015 to identify papers published since the initial search. A sensitive search strategy was designed in consultation with an information science specialist (CF) using both subject headings and text terms and comprised a combination of three facets: professional behaviour change; theory, framework or technique; and interventions. The search strategies used are detailed in Additional file 1. Reference lists of included papers were screened for additional papers as were articles known to the review team. Our search started in 1996 as this was consistent with the introduction of the evidence-based medicine movement [12], and an associated increase in evidence-to-practice related publications [13].

Papers were included if two criteria were met: (1) the paper described a method (process, tasks, approach) for designing interventions to change HCPs’ behaviour or practice, and (2) the primary focus of the paper was on the intervention design process (as opposed to, e.g. on intervention evaluation). We defined interventions as: ‘a method or technique designed to enhance adoption, implementation and sustainability of a clinical/therapeutic program or practice, a specific clinical/therapeutic practice or delivery system/organizational arrangement being tested or implemented to improve healthcare outcomes’ [14]. A HCP was defined as any member of the healthcare team providing care, and their behaviour was defined as objectively observable actions (as opposed to, e.g. their knowledge or reasoning).

Protocol papers were included if the primary aim of the protocol was to describe intervention design methods or process. Papers were excluded if they pertained to HCPs’ behaviours not related to their clinical practice (e.g. HCPs’ eating healthily, exercising). While papers that report the implementation and evaluation of interventions may include descriptions of how the intervention was designed, this is rarely in a detailed and replicable manner [15]. As our interest was in providing a resource to guide researchers in the process of intervention design, we excluded papers that lacked enough detail for replication. These decisions were made based on the consensus of the three reviewers (HLC, JES, NK). Due to resource limitations, we also excluded articles that were not in English.

A screen of titles and abstracts was conducted by one rater independently (shared by HLC, JES, NK) and was followed by a review of full papers by three raters independently (HLC, JES, NK). An interrater reliability analysis using the Kappa statistic was performed to determine consistency among raters for the full-text review.

For all included full texts, general descriptive information (authors, year, journal, name of method if so named) were extracted and tabulated. To extract and analyse data about the methods, a two-stage process was carried out. Stage 1 involved generating a framework for data extraction and analysis. Three reviewers (HLC, JES, NK) progressed in iterative cycles of reading and discussing the included papers to identify similarities and differences between them and used these discussions to develop a list of items to be extracted. The iterative cycles were continued until an agreement between the three authors was reached. In part, this process was necessary to improve our understanding of the tasks that constitute intervention design and allow us to extract data outside of simply a brief description of the method. The resulting descriptive variables to be extracted were believed to be the most critical, and it included a brief description of the design tasks. Data extraction was conducted by two individuals (HLC, JES) independently first followed by consensus discussions for discrepancies (Stage 2).

## Results

**Fig. 1:**
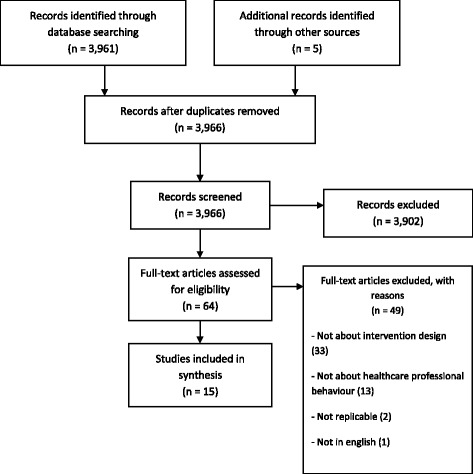
PRISMA diagram

Prior to de-duplication there were 4667 records (MEDLINE 1512, Embase 1567, PsycINFO 1588). Once the duplicates were removed, we had 3966 citations to screen (Fig. 1). We excluded 3902 records based on the title and abstract screen resulting in 64 articles assessed for eligibility with a full-text screen. Following full-text review and consensus discussion, 49 articles were excluded leaving a total of 15 articles in the review. Reasons for exclusion based on a full-text review included papers that were not about intervention design (*n* = 33), not about HCPs’ behaviour (*n* = 13), not enough detail for replication (*n* = 2), and not in English (*n* = 1). The mean Kappa statistic across all pairs was .43 indicated moderate agreement [16].

The stage 1 process of data extraction resulted in four categories for extraction:

1. The context in which the method was developed; either generic (described) or specific (i.e. behaviour, providers, setting, clinical condition—described as able).
2. The level of change that the method was focused on (i.e. individual, organization, system, other).
3. Whether the method incorporated any other type of published approach, tool, or resource as a component of the design process (e.g. incorporated the Theoretical Domains Framework (TDF) [17] as part of the process).
4. A brief overall description of the tasks included in the method, if the method included barrier identification, if it included a process of component selection that linked barriers to intervention components, the use of theory at any stage of the design process, and whether users were engaged in intervention design (i.e. was input sought regarding feasibility or acceptability of the intervention from the potential targets for behaviour change).

### General descriptive information about the papers

Table 1 provides a summary of the included 15 papers that were published between 2001and 2014. The 15 papers were published in five journals with eight in Implementation Science, three in BioMed Central Health Services Research, and two in Quality and Safety in Health Care. Three of the 12 papers reported a formal name or label for the method: Analysis, Development, Design, Implementation, Evaluation (ADDIE) Method [18], the Quality Enhancement Research Initiative (QUERI) [14], and the Theoretical Domains Framework Implementation (TDFI) approach [19]).

**Table 1:** Summary of intervention design methods and key characteristics

No., author, year (label if given)	Method summary	Generic or specific (described) context	Level of change	Builds on other methods, tools yes/no (defined)	Barrier identification yes/no	Links barrier to components yes/no	Uses theory yes/no	Input from users yes/no
1. Battles, 2006[18] (ADDIE method)	1) Analysis: identify the target and needs, 2) Development: define behaviour, and learning outcomes and sequence, 3) Design: specify content and medium of prototype, 4) Implementation 5) Evaluation	Patient safety	Ind	No	Yes	Yes	Yes	No
2. Cabassa, 2001[23]	1) Researchers/stakeholders review and modify intervention 2) Ensure acceptability (focus groups) 3) Modify intervention, plan implementation and training 4) Pilot for feasibility, acceptability, initial effects 5) Rigorous evaluation	Providers: case managers Setting: outpatient mental health Clinical condition: serious mental health and cardiovascular disease	Other	Yes (IM, Participatory Action methods)	No	No	Yes	Yes
3. Chandler, 2014[30]	1) Formative research (observations, interviews, focus groups) with targets 2) Review existing literature for behaviour change strategies and theories 3) Face to face workshop with researchers and experts to review results of first two steps and propose potential theory-based intervention strategies 4) Design intervention 5) Pilot and pre-test, and determine final intervention	Behaviour: use of malaria rapid diagnostic test and adherence to test results Provider: Tanzanian community health workers and nurses Setting: Tanzanian community; low resource settings Clinical condition: malaria	Ind	No	Yes	Yes	Yes	No
4. Clyne, 2013[24]	1) Development: Literature review of empirical and theoretical evidence to inform potential intervention components. Organise influencing factors from the literature using a model of potential influencing factors (PRECEDE model). 2) Use consensus based methods, case studies, and focus groups to develop and confirm appropriate actions by GPs (treatment algorithms for best practices for prescribing). 3) Finalize intervention, pilot test and conduct interviews with targets regarding feasibility and acceptability of intervention	Behaviour: decreasing inappropriate prescribing in older adults Provider: general practitioners Setting: primary care in Ireland Clinical condition: older adults	Ind	Yes (MRC)	Yes	No	No	Yes
5. Curran, 2008 [14] QUERI method	1) Determine site-specific needs/barriers (observations and interviews) 2) Develop the intervention with staff, research experts, and clinical experts (meetings, consulting with others, facilitation by local champions)	Behaviour: implementing a guideline for depression Provider: all staff Setting: substance abuse clinic Clinical condition: depression/substance abuse	Ind Org Sys	Yes (Stetler’s concept of formative evaluation, Rubenstein’s EBQI)	Yes	Yes	Yes	Yes
6. Foy, 2007[28]	1) Specification of target behaviours 2) Select theoretical framework 3) Conduct predictive study 4) Choose variables to target 5) Map variables to behaviour change techniques 6) Choose method of delivery 7) Operationalise intervention components	Behaviour: implementing disclosure behaviours for diagnosing dementia Providers: mental health teams Clinical condition: dementia	Ind	Yes (BCT)	Yes	Yes	Yes	Yes
7. French, 2012[21]	1) Who needs to do what differently? 2) Using a theoretical framework, which barriers and enablers need to be addressed? 3) Which intervention components could overcome the modifiable barriers/enablers? 4) How can the behaviour change be measured and understood?	Behaviour: implementing better back pain care Providers: GP’s Clinical condition: low back pain	Ind	Yes (TDF, BCT)	Yes	Yes	Yes	Yes
8. Fretheim, 2004[32]	1) Researchers engage in structured reflection 2) Review relevant evidence 3) Conduct a survey of the targets 4) Engage targets in discussion while piloting the intervention	Behaviour: implementing improved pharmacological management Providers: GP’s Clinical condition: hypertension and hypercholesterolaemia	Ind	No	Yes	Yes	No	Yes
9. Kolehmainen 2012 [22]	1) Identify behaviour change techniques 2) Providers generate evidence-based and context-relevant modes of delivery for the techniques (advisory team and brainstorming) 3) Use theory and BCT taxonomy to create hypotheses about the mechanisms of change	Behaviour: improved caseload management through three specific goal-setting behaviours Provider: pediatric occupational therapists	Ind	Yes (BCT, MRC)	No	Yes	Yes	Yes
10. McDermott, 2010[31]	1) Identify the intervention using evidence and theory 2) Conduct interviews with targets re factors likely to influence 3) Analyse and modify the intervention 4) Conduct ‘think aloud’ interviews with targets re intervention 5) Finalize intervention	Behaviour: implementing guidelines Provider: general practitioners Setting: General practices Clinical condition: stroke and respiratory tract infection	Ind	No	Yes	Yes	Yes	Yes
11. Porcheret, 2014[25]	1) Clearly define desired change and targets for change 2) Analyse current practice including barriers and facilitators using a structured theory-based approach—TDF 3) Determine intervention strategies based on a taxonomy of behaviour change techniques and theory—Adult Learning Theory 4) Implement 5) Evaluate	Behaviour: enhanced consultation by GP’s for people with OA according to guidelines Provider: general practitioners Setting: primary care Clinical condition: osteoarthritis	Ind	Yes (Implementation of Change Model [41], TDF and BCT)	Yes	Yes	Yes	Yes
12. Sassen, 2012[26]	1) Needs assessment of the population 2) Define performance objectives as they relate to determinants (change objectives) 3) Link the performance objectives to the determinants and suggest intervention methods to change the determinants that are based on theory 4) Develop and pre-test the intervention 5) Adopt, implement, and sustain intervention	Behaviour: encourage patients to engage in physical activity Provider: nursing or physiotherapy health professionals Setting: cardiovascular inpatient and outpatient care Clinical condition: patients with cardiovascular disease	Ind	Yes (IM)	Yes	Yes	Yes	Yes
13. Schmid, 2010[27]	0) Complete a needs assessment by conducting interviews with providers 1) Set performance objectives that are linked to related determinants and expected change 2) Select a theory-based intervention methodology to determine intervention components that are linked to the objectives set in step 1 3) Design the intervention using all IM steps, and the results of the needs assessment from step 0 4) Tailor the resulting intervention to local needs, adopt, and implement 5) Monitor and evaluate	Behaviour: adherence to stroke prevention guidelines Provider: entire stroke team physicians, nursing, allied health Setting: continuum of stroke care Clinical condition: Stroke	Ind	Yes (IM)	Yes	Yes	Yes	Yes
14. Taylor, 2013[19] (TDFI)	1) Engage stakeholders 2) Identify target behaviours 3) Identify barriers and facilitators using TDF, focus groups, barrier questionnaire 4) Engage stakeholders to develop local strategies linked to barriers based on BCT plus current literature 5) Support stakeholders to implement 6) Assess feasibility and acceptability	Behaviour: adhere to guideline for reduce risk of feeding into misplaced nasogastric tubes Provider: setting: acute care Clinical condition: in-patients with nasogastric tubes	Ind	Yes (TDF, BCT)	Yes	Yes	Yes	Yes
15. van Bokhoven 2004 [20]	1) Identify problem/target for improvement 2) Problem analysis 3) Design the intervention 4) Pre-test 5) Adopt and implement 6) Evaluate	Quality improvement	Ind	Yes (IM)	Yes	Yes	Yes	Yes

*Ind* Individual, *Org* Organization, *Sys* System, *Other* Focus on intervention adaptation, *ADDIE* Analysis, Development, Design, Implementation, Evaluation, *IM* Intervention Mapping, *GP’s* General Practitioners, *MRC* Medical Research Council guidance on the evaluation of complex interventions, *QUERI* Quality Enhancement Research Initiative, *EBQI* Evidence Based Quality Improvement, *BCT* Behaviour Change Technique Taxonomy, *TDF* Theoretical Domains Framework, *TDFI* Theoretical Domains Framework Implementation

### Contexts, target levels, and incorporating other processes/steps/tools/resources

All 15 included papers specified a context in which the method was initially developed but indicated that the method could be used outside of the particular context; indeed, this was the purpose of the papers. Examples ranged from broad contexts such as quality improvement [20] or patient safety [18] to specific contexts such as general practitioners’ behaviours for the treatment of low back pain [21] or occupational therapists’ caseload management [22]. Thirteen of the 15 papers proposed methods targeting individual HCPs; one of these [22] proposed methods targeted at the team level but not the organization. Only one paper targeted change across individual, organization, and system levels [14]. The remaining paper focused on the feasibility of the intervention and not on the change at a specific level per se [23].

Eleven of the 15 papers incorporated other approaches, tools, or resources as a component of the intervention design process [14, 19–28]. Four of these [20, 23, 26, 27] incorporated Intervention Mapping [29], another three incorporated both the TDF and the BCT taxonomy [19, 21, 25], two incorporated the BCT taxonomy without also incorporating the TDF [22, 28], and two incorporated the Medical Research Council (MRC) framework [22, 24]. Five of 15 incorporated just one other published tool [20, 24, 26–28], and one incorporated three published tools [25].

### Design tasks in the methods

All of the 15 identified papers included a number of tasks required for the design process. These steps ranged from two [14] to seven [28] with a median of 5 tasks.

All but two [22, 23] of the papers included some form of barrier identification. One of these papers [22] reported an assumption that barriers had already been identified in previous work and provided a method for linking barriers to intervention components. The other paper [23] focused on adapting an intervention using stakeholder engagement and did not address barrier identification specifically. In the papers where barrier identification was covered, the methods included observations, interviews and/or focus groups [14, 18, 30–32], surveys [32], literature reviews [24], structured reflection by the researchers [32], job analysis and expert consensus [18], and undertaking a predictive study to identify factors influencing the behaviour [28]. Six papers used the structured interview processes outlined in the TDF [19, 21, 25] or in Intervention Mapping [20, 26, 27].

All but two papers [23, 24] included linking barriers to intervention components. As mentioned, one of these papers [23] focused on adapting an intervention using stakeholder engagement and did not address linking to intervention components specifically. The second paper [24] did conduct a barriers assessment but did not describe how barriers were linked to intervention components. Methods to link barriers to intervention components included mapping TDF-barriers to the BCT taxonomy [19, 21, 25], as well as using the structured approaches described in Intervention Mapping [20, 26, 27]. One paper used what they termed ‘development panels’ which involved staff, research experts, clinical experts, and local champions participating in a range of meetings and consultations [14]. All but two papers [24, 32] included the use of theory. There were papers (e.g. [21]) that used broad theoretical frameworks by way of incorporating pre-existing approaches that had used theory in their development (e.g. the TDF [17]). Other papers used more specific, discrete theories chosen based on the context in which the specific intervention was being developed. Examples included using Social Cognitive Theory [33] to design a computer-delivered intervention to enhance the use of practice guidelines in general practices [31], and using theories of risk perception to improve physical activity in cardiovascular patients as part of the Intervention Mapping process [26]. All but two papers [18, 30] included some approach to gathering input on the design of the intervention from the users or the individuals that were the target of the intervention. In all cases, this involved testing, piloting, or showing the intervention to the targets and gathering feedback in the form of discussion or interview. In two cases, this included formal cognitive interviews [28, 31].

## Discussion

We conducted a systematic review of methods for designing interventions to change HCPs’ behaviour. We found 15 papers that outlined 15 methods. All of the papers reported methods that were developed within a specific context. Thirteen papers targeted only individual HCPs with one paper targeting change across individual, organization, and system levels. The methods consisted of at least two tasks and, at most, seven tasks. Thirteen papers included some form of barrier identification and 13 provided direction for linking barriers to intervention components; however, these were not the same 13 papers. Thirteen papers included the use of theory, and another 13 included gathering input on the design of the intervention from the targets of the intervention.

A number of publications related to designing interventions were not included in this review, For example, MRC guidance documents for developing and evaluating complex interventions [9], publications outlining the KT process [34, 35], tools, and frameworks that examine barriers assessments for KT intervention [17], and taxonomies of behaviour change techniques [10]. While these publications are certainly of high relevance to intervention development and evaluation process in general, these were not included here as they were all judged to provide limited detail about the specific, replicable actions to *design* interventions. For example, the MRC guidance emphasises the importance of designing interventions, but is limited in concrete guidance on how to actually do this in practice. In addition, a number of papers were identified that specifically stated intervention design as an aim [36, 37] but that lacked the detail that would allow replication. It could be that there are additional papers not included in this review that could facilitate intervention design. The majority of the methods found (11/15) incorporated other tools or resources, albeit not in identical ways. While we do not know the rationale for doing so, it could be that existing tools alone are felt to be inadequate for intervention design. Future studies on additional design methods that incorporate other existing tools and resources will likely aid in advancing methodologies for designing interventions.

There were two additional papers not included in our review that received significant dialogue during consensus and therefore warrant some discussion. One of these was Eccles et al. [38] which includes a description of using a theory to design a KT intervention. We felt this paper provided a rationale and description of conceptual issues related to using theory to develop an intervention; however, the degree of detail on *how* to design an intervention in this paper is limited. The second paper warranting discussion was on Intervention Mapping [29]. This paper outlined a method for intervention design for health behaviours, not for HCPs’ behaviours, and was therefore excluded. However, we did find four methods papers for designing interventions to change HCPs’ behaviour that incorporated Intervention Mapping [20, 23, 26, 27]. It is likely that other methods to design interventions to change health behaviours could similarly be adopted to design interventions to change HCPs’ practice.

Two main gaps seem evident in our review of the intervention design literature. First, limited methods target change in organisations or systems, or at least were developed with a focus on the organization or system. We found only one study [14] that did this explicitly. A second study [22] targeted teams as well as individuals but did not do so at the organizational level. We found limited methods to specifically take the organisation and system level contexts into consideration, as well as methods that consider all levels. While it is true that many of the methods’ approaches to barrier identification could result in a focus on the organisation or system should barriers at these levels be identified (for example, see French et al. [21]), the implicit focus of these papers targeted individual behaviour change. Future studies should consider how and under what circumstances to ensure that organisational and system level change is considered.

The process of undertaking this review highlights a second gap: the need for a better understanding of what activities constitute intervention design. Our iterative process of determining the intervention design variables for extraction and the subsequent extraction of those variables has led to a better understanding of the steps inherent in intervention design, at least according to current methods. There appear to be four steps common to intervention design: barrier identification, linking barriers to intervention component selection, use of theory, and user engagement (i.e. seeking input on feasibility or acceptability of the intervention from the potential targets). While we do not necessarily understand the best order for these tasks nor do we know what additional tasks are required, it does represent a simple structure of potential prototypical steps for the design of a KT intervention. Additional understanding of these tasks, as well as a more in-depth consideration of potentially additional tasks that should be but are not yet routinely adopted, would improve intervention design methods.

Almost all of the methods found (13/15) used theory at some point in the tasks for intervention design, yet evidence indicates theory is rarely used in the design of interventions, or at least rarely reported [39, 40]. Selecting one of these published methods or building on these methods is likely to guide researchers to use theory. Our review did not measure the degree to which these methods are used but this would be a useful future area of research. Additionally, future methodological work could focus on best practices for the use of theory to design an intervention.

Several limitations of this review warrant discussion. We used only one rater for the title and abstract review. Although support exists for the validity of using one rater [41], having two raters would have reduced the possibility of omitting a potentially relevant study. All of the included methods were developed specifically for healthcare environments. This could be in part due to our search strategy. Other methods from disciplines outside of healthcare could yield additional and suitable methods, as could methods developed prior to 1996. Several limitations exist that might have reduced the number of potential methods found. Due to the challenges in adequately searching books, we did not include books or book chapters in our search. Our inclusion criteria meant that we did not include any studies that reported on the testing of an intervention in addition to the development of the intervention. In part, we did this to isolate methods that were described in enough detail to be able to replicate and adequately guide the design of an intervention. Lastly, we did not search grey literature, making the review susceptible to publication bias [42], and we only chose to use three databases. It is feasible that additional methods exist.

## Conclusions

This systematic review outlined 15 published and replicable methods for designing interventions to change HCPs’ behaviour. Its use as a resource and as a catalyst for improved quality and quantity of methods is encouraged. Although these methods included varied steps, there was a general agreement that designing an intervention for individual-level change includes identifying barriers, selecting intervention components, using theory, and engaging end-users. Methods for designing organisation and system-level interventions were limited. Further comparative analysis of how the common tasks are completed in the different methods will provide a starting point for developing more detailed guidelines for designing KT interventions. Future research should focus on the degree to which these methods have been used, determining how such methods could be better adopted and further development of both guidance for the existing methods and, potentially, new methods.

## Additional file

Additional file 1: Intervention Development: Search strategies. (DOCX 16 kb)

## Abbreviations

ADDIE: Analysis, Development, Design, Implementation, Evaluation Method
TDFI: Theoretical Domains Framework Implementation Approach
HCP: Healthcare professional
KT: Knowledge translation
MRC: Medical Research Council
QUERI: Quality Enhancement Research Initiative
TDF: Theoretical Domains Framework
