# Supplementary material for: Methods for designing interventions to change healthcare professionals’ behaviour: a systematic review
Source: Implement Sci. 2017 Mar 4;12:30. doi: 10.1186/s13012-017-0560-5 (PMC5336662; doi:10.1186/s13012-017-0560-5)
Supplement: Additional file 1: — Intervention Development: Search strategies. (DOCX 16 kb) [file 13012_2017_560_MOESM1_ESM.docx]

**Additional file 1**

**Intervention Development: Search strategies**

**Database: Embase <1996 to 2015 Week 16>, Ovid MEDLINE(R) without Revisions <1996 to April Week 2 2015>, Ovid MEDLINE(R) In-Process & Other Non-Indexed Citations <April 20, 2015>**

***Ovid Multifile Search URL: https://shibboleth.ovid.com/***

1 Physician's Practice Patterns/ use medf

2 exp Evidence-Based Practice/ use medf

3 (chang$ adj3 (behavio?r or practice)).tw.

4 (professional adj3 (practice or behavio?r)).tw.

5 evidence based.tw.

6 implementation.tw.

7 or/1-6

8 (develop$ or design$).ti.

9 (theory or theories or theoretical).ti.

10 (mechanism? or approach$ or method$).ti.

11 (framework or constructs or process$).ti.

12 (tools or technique$ or content or strateg$).ti.

13 or/8-12

14 intervention?.ti.

15 (intervention? adj3 (chang$ or design$ or develop$ or behavio?r)).tw.

16 intervention study/

17 or/14-16

18 7 and 13 and 17

19 18 not conference abstract.pt.

20 remove duplicates from 19

**PsycINFO 20^th^ April 2015**

***URL: www.ebscohost.com/***

S1 .DE "Evidence Based Practice"

S2 .TX ( (chang* n3 (behavio#r or practice)) ) OR TX ( (professional n3 (practice or behavio#r)) ) OR TX evidence based OR TX implementation

S3 .S1 OR S2

S4 .TI ( (develop* or design*) ) OR TI ( (theory or theories or theoretical) ) OR TI ( (mechanism* or approach* or method*) ) OR TI ( (framework or constructs or process*) ) OR TI ( (tools or technique* or content or strateg*) )

S5 .TI intervention* OR TX ( (intervention* n3 (chang* or design* or develop* or behavio#r)))

S6 .DE "Intervention

S7 .S5 OR S6

S8 .S3 AND S4 AND S7

S9 .S3 AND S4 AND S7 Limiters - Publication Year from: 1996-2013
